# Hierarchical multicolor nano-pixel matrices formed by coordinating luminescent metal ions to a conjugated poly(4′-octyl-2′,6′-bispyrazoyl pyridine) film via contact printing

**DOI:** 10.1038/srep08406

**Published:** 2015-02-12

**Authors:** Supratim Basak, Md Ahamad Mohiddon, Martin Baumgarten, Klaus Müllen, Rajadurai Chandrasekar

**Affiliations:** 1 Functional Molecular Nano-/Micro-Solids Laboratory, School of Chemistry, University of Hyderabad, Prof. C. R. Rao Road, Hyderabad – 500046, India; 2 Max Planck Institute for Polymer Research, Ackermannweg 10, D-55128, Mainz, Germany

## Abstract

We introduce a cost-effective, yet feasible reactive printing approach namely, “coordination chemistry at the conjugated ligand polymer surface”. By using a contact printing technique we selectively fabricated high resolution nano-pixel configurations consisting of red, blue and green (R,G,B) colors arranged in a hierarchical three-dimensional fashion. For this, we have synthesized a novel blue emitting conjugated ligand polymer, [poly(4′-octyl-2′,6′-bispyrazoyl pyridine)] (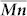 ~ 7–8 KDa) having a tridentate ligand in its repeating unit which forms a thin film prone of binding metal ions. On top of this ligand polymer film, a layer of high-resolution cross-stripes (width ca. 800 nm) was printed comprised of Eu and Tb ions over a large area ~ 100 × 100 μm^2^. The final woodpile-like assembly produced a new pixel group consisting of B; BG; BR; and BGR (White) colors. The area of the white emitting square is ca. 0.64 μm^2^. The patterned layers that make up the pixels are very thin and thus this new technique might find applications in flexible light emitting devices.

Modern technologies aiming to excel the performance of multimedia display devices continue to be in great demand^1^,^2^. One of the innovative solutions is the development of technological know-how to achieve multicolor lights from the emissive organic layer composed of well-ordered thin matrix pixels down to nano regime^3^,^4^. However, the construction (deposition and patterning) of color matrix pixels in a nano scale geometric layout is still a challenging and pertinent task. In commercial devices, both red, green, blue (RGB) and RGB white (RGBW) pixels are typically used with the cross section in the range of several microns. Compared to devices having traditional RGB stripe pixels, the inclusion of white pixels in the RGBW matrix boosts the brightness of the images and consumes less power with increasing contrast^5^. In order to realize extreme high resolution in the display devices, increasing the number of pixels by reducing the pixel sizes is an innovative and demanding strategy.

Nano-pixel matrices composed of light emitting molecules or polymers can be created by using top-down lithographic patterning techniques using X-ray^6^,^7^, electron beam^8^, and photolithography^9^, although none of them allows an economical scaling up. On the contrary, a soft lithography procedure allows a rapid patterning of large surface areas with multiple luminescent components. Therefore, soft lithography techniques^10^, such as micro contact (μ-CP) and micro transfer printing, have become most feasible tools for the fabrication of OLEDs^11^,^12^. To pattern light emitting polymers, CP is the preferred technique owing to the possibility to cover large surface areas with submicro-scale spatial resolution and elimination of vacuum processes. In this method, the polymer ink is transferred to the surface using a rigid or elastomeric mold. The use of conjugated polymer blends to achieve active layers suffers from phase separation due to incompatibility of dissimilar polymer mixtures^13^. Moreover, controlling the length and direction of the phase separated polymer mixture to form a high resolution pattern is quite difficult. In order to achieve high spatial resolution, the deterioration of pattern features by lateral diffusion of ink after the transfer of the ink on to the surfaces must be prevented. Existing solutions include using (i) reactive μ-CP^15^, (ii) a polymer ink that interacts strongly with the surfaces through electrostatic interactions^14^, and (iii) heavy weight dendrimers to suppress lateral diffusion^16^, but none of them have been implemented for light emitting applications. From this viewpoint an original chemical protocol is needed to develop lithographically patterned stable multicolor nano-pixels. To the best of our knowledge there is still no reactive μ-CP methodology known based on “*coordination chemistry at the conjugated ligand polymer film surface*”^17^,^18^.

We envisaged the application of a versatile CP strategy for the reactive modification of a layer of blue emissive conjugated ligand polymer by selective coordination with luminescent metal ions (Red: Eu^3+^ and Green: Tb^3+^). Such a technique would allow the creation of high resolution multicolor geometric layouts which include white (layered RGB) pixels down to nano domain. To implement this strategy, the development of a novel synthetic procedure for polymerization of an organic ligand molecule is necessary. It is known that the tridentate 2,6-bispyrazoyl pyridine (bpp) unit can bind hexacoordinated lanthanide complexes to form photoluminescent complexes with coordination number nine^19^. In order to exploit bpp ligand based conjugated polymer film as a platform to print photoluminescent metal ions to generate multicolor nano-pixels, we have homo polymerized 4′-octyl-2′,6′-bispyrazoyl pyridine ligand^18^. In the resultant polymer the bpp core helps to coordinate photoluminescent metals while octyl chain units provide easy solution processability.

In this letter we report a unique method to reactively coordinate photoluminescent metal ions to a novel blue emitting poly(4′-octyl-2′,6′-bispyrazoyl pyridine) (**Poly**) film surface through the CP technique (Fig. 1). The surface of the blue emitting polymer is cross stripe patterned by coordinating red luminescent Eu(III) and green luminescent Tb(III) inks (at a right angle 90°) to obtain a well ordered woodpile like multicolor matrix pixel arrangements. The final woodpile structure consists of four different areas which differ in their chemical composition and thereby form a remarkable four color pixel group consisting of : (i) blue: from the emitting polymer, (ii) purple: from the mixing of polymer and Eu(tta)_3_ emissions, (iii) pink: from the mixing of polymer and Tb(acac)_3_ emissions and (iv) white: from the mixing of polymer, Eu(tta)_3_ and Tb(acac)_3_ emissions.

## Results and Discussions

### Syntheses of monomer, dimer, and homopolymer of bpp

**Figure 2:**
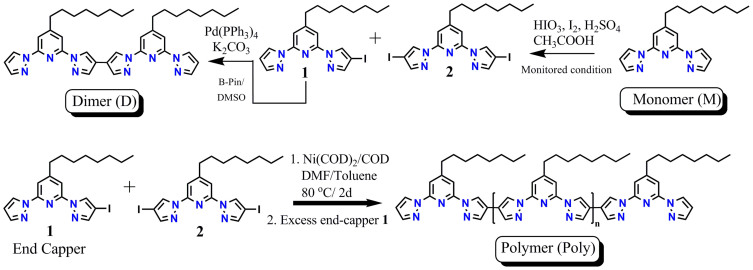
Polymer Synthesis. Syntheses of monomer (**M**), dimer (**D**) and of 4-octyl-2,6-di(1H-pyrazol-1-yl)pyridine polymer (**Poly**).

At first, to probe the effect of conjugation on the optical properties of the homopolymer (**Poly**), n-mers such as monomer (**M**), and dimer (**D**) were synthesized. **M** was prepared by our reported procedure starting from a low cost citrazinic acid in seven synthetic steps^18^. For the preparation of **D**, a controlled iodination of **M** was performed to get monoiodinated (**1**) (24%) and diiodinated (**2**) (99%) compounds (Fig. 2)^18^. Afterwards compound **1** was homocoupled under Suzuki conditions using bis(pinacolato)diboron, Pd(PPh_3_)_4_ and K_2_CO_3_ to get dimeric bpp (**D**). The homopolymer of bpp (**Poly**) was synthesized via a Yamamoto reaction of **2** using the 2-(4-iodo-1H-pyrazol-1-yl)-4-octyl-6-(1H-pyrazol-1-yl)pyridine (**1**) as an end-capper (Fig. 2). The polymerization reaction was performed using Ni(COD)_2_/COD/bipyridine catalysts in a mixture of dimethylformamide and toluene by stirring at 80°C. At the end of the reaction an excess of compound **1** was added with the objective to maximize end-capping of polymer. The number averaged molecular weight 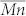 of the polymer as revealed by gel permeation chromatography analysis is in the range of 7–8 KDa (number of repeating units; *n_avg_* = 22). The thermal decomposition temperature (*T*_D_) with 5% weight loss of the polymer is 380°C (Fig. S1).

### Solid state structure of model dimer compound

**Figure 3:**
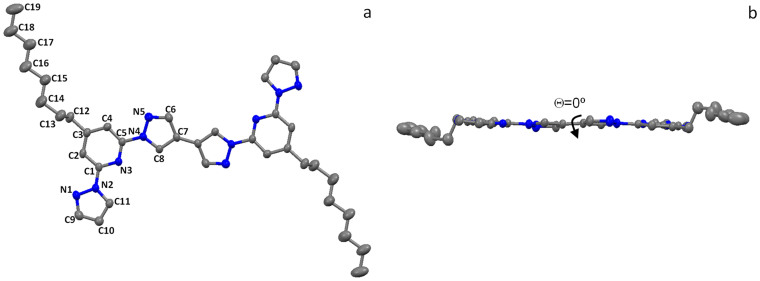
Single crystal structure of dimer (D) at 293 K. (a), top view. (b), side view. Hydrogen atoms are omitted for clarity (nitrogen atoms are shown in blue color).

The single crystal structure of the dimer (**D**) revealed that the orientation of the two bpp units was planar with a 0° torsion angle between the two connecting (C7–C7′) pyrazole rings (See Fig. 3). This observation is indicative of the expected efficient electronic conjugation amongst the bpp repeating units of the homopolymer (**Poly**).

### Optical properties of monomer, dimer and polymer in solution and solid state

**Figure 4:**
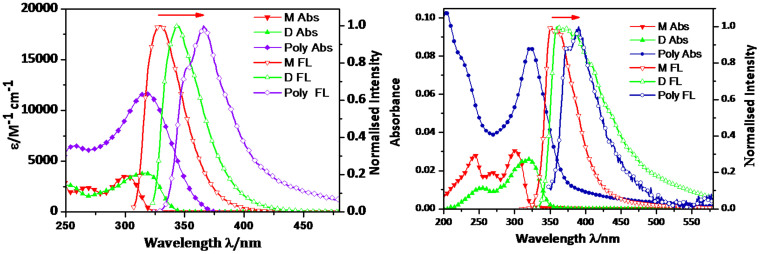
Optical Studies. Absorbance and emission spectra of monomer (**M**), dimer (**D**) and polymer (**Poly**) in (a) solution and (b) solid states.

The photo physical characteristics of the monomer, dimer, and polymer were studied both in CHCl_3_ solution and in the solid state; all spectroscopic data are summarized in Table S1 (supporting information). The solution state absorbance and emission spectra of **M**, **D** and the **Poly** are shown in fig. 4a. In solution state **M**, **D** and **Poly** exhibited absorption maxima at 301/268, 317/253, 316/258 nm due to π-π* transitions and emission maxima at 330, 344, 366 nm, respectively. The polymer showed a low energy emission maximum at 366 nm because of increased electronic conjugation leading to a larger band gap of ca. 3.38 eV compared to monomer. In the solid state the absorption spectra of **M**, **D** and **Poly** showed slightly red shifted broad band maxima positioned at 301, 320, and 323 nm, respectively (Fig. 4b). Further the solid state fluorescence emissions of **M**, **D** and **Poly** displayed largely red shifted emission maxima at 351, 361 and 390 nm compared to their solution states indicating solid state aggregation and pronounced interchain interactions^21^.

### Woodpile-like contact printing on the polymer surface with Eu(III) and Tb(III) complexes using coordination interactions

As the synthesized polymer comprised a series of tridentate ligands for metal coordination and also forms fine blue emitting films, the thin polymer film was employed as a reactive template for CP. To perform CP elastomeric polydimethylsiloxane (PDMS) stamps having rectangular shape arrays were prepared from the master (NT-MDT AFM test gratings model TGZ3) by the established procedures^22^. In view of the polarity of the ink molecules and the wettability of the PDMS stamp, a 1:1 mixed solution of ethylene glycol and acetone was used to prepare inks containing Eu(III) and Tb(III) metal ions. Ethylene glycol makes the ink viscous and acetone adequately wet the PDMS surface facilitating surface coordination reaction at the interfaces between stamp and polymer layer to form high resolution stripes. Hence the PDMS surface does not have to be activated by oxidation through an oxygen plasma treatment. The red luminescent ink was prepared by dissolving 1.5 mg of Eu(tta)_3_ in 1 mL of mixed solution. At first a thin film of ligand polymer layer was generated on a clean silica substrate by spin coating (at 1000 rpm/10 sec/6000 rpm/10 sec) of a CHCl_3_ solution of polymer (c ~ 4 mg/mL). The stamp was carefully wetted with the ink solution and the excess solution was removed by using a filter paper. Then the stamp coated with ink was printed on the thin polymer film substrate and subsequently a weight of 20 g (area: 2 × 1.5 cm metal block) was kept on top of the stamp to make a uniform smooth contact between the bottom of the stamp and the polymer substrate. After an overnight reaction the stamp was lifted off very carefully to avoid any mechanical damage.

**Figure 5:**
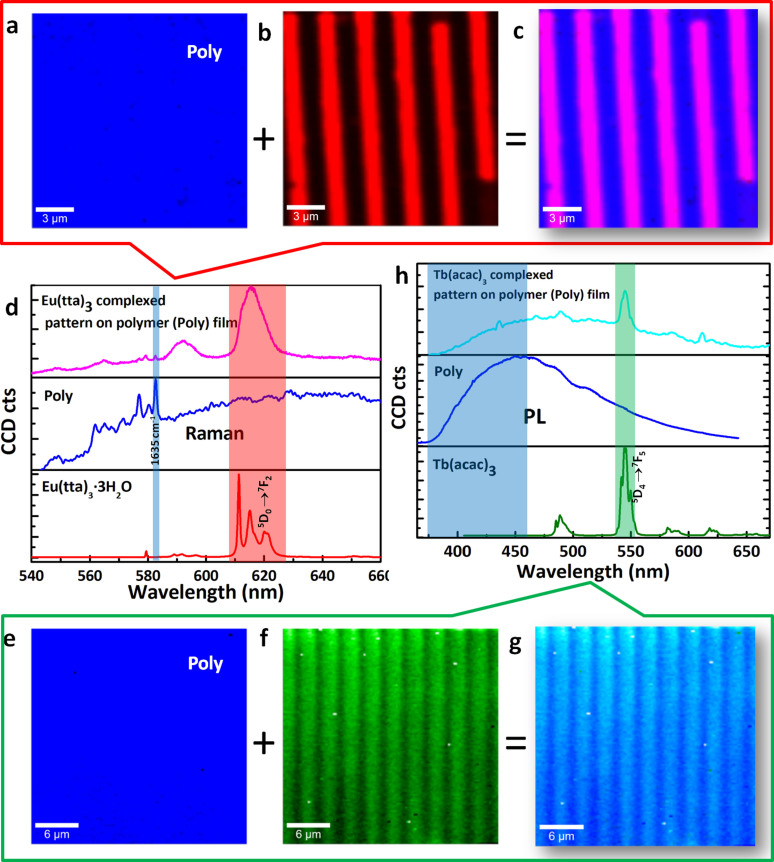
Patterning of Eu(III) and Tb(III) inks coordinatively on a polymer surface. Raman/PL images of Eu(tta)_3_ printed stripes on the polymer surface: (a), False color Image of 1635 cm^−1^ Raman peak corresponding to blue PL of polymer. (b), False color Image of the red PL peak (610–620 nm) from stripes. (c), Combined image of (a) and (b). (d)), Spectra of Eu(tta)_3_ complexed pattern on polymer surface, polymer and Eu(tta)_3_·3H_2_O, the marker peaks are shown in violet and magenta color for polymer and Eu(tta)_3_, respectively. PL images of the Tb(acac)_3_ pattern on polymer surface: (e), False color Image of a peak in the region between 376–472 nm corresponding to blue PL of polymer. (f), False color Image of the green PL peak in the region between 540–550 nm from stripes shown in green color. (g), Combined image of (e) and (f). (h), Spectra of Tb(acac)_3_ complexed pattern on polymer surface, polymer and Tb(acac)_3_·H_2_O, the marker peaks are shown in violet and green color for polymer and Tb(acac)_3_·H_2_O, respectively.

The chemical composition of the patterns was confirmed by performing micro Raman/photoluminescence (PL) spectroscopy and imaging on a laser confocal microscope (LCM) equipped with a 532 nm neodymium-doped yttrium aluminium garnet (Nd/YAG) and 355 nm ultra violet (UV) diode lasers. For the local laser excitation of the sample containing Eu(III) patterns on the polymer, a 532 nm visible laser was used. The collected signals from the sample were filtered using a 532 nm long pass edge filter (LPEF) and sent to a CCD detector. In the sample, the polymer has no direct electronic absorption in the visible wavelength range, while the Eu(tta)_3_complex has a slight absorption in this range. As a result a clear Raman signal from the polymer and red PL signal (*f*-*f* transitions) from the Eu(tta)_3_ patterns was obtained and subsequently used for 2D mapping. Imaging of the high intensity Raman signal of the polymer appeared at 583 nm (1635 cm^−1^; C = C stretch) is shown in blue color corresponding to the pure blue emitting polymer film (Fig. 5a and 5d). PL Imaging of the 610–625 nm signal region (^5^*D*_0_ →^7^*F*_2_) from the red emitting Eu(III) exhibited the formation of micro stripes at a regular 1.5 μm intervals on the polymer surface (red color, Fig. 5b and 5d). To see the mixed colors, the two images (Fig. 5a and 5b) were superimposed and shown in fig. 5c. Here, the blue areas indicate the polymer and the pink stripes (red + blue color mixing) correspond to Eu(tta)_3_ stripes, their interface [**Poly**(Eu(tta)_3_)_n_] and unreacted polymer as well.

Using the same protocol a series of green emitting Tb(acac)_3_ stripes at regular gaps was printed with high accuracy on the reactive polymer surface. The green ink solution was prepared by dissolving 2.12 mg of Tb(acac)_3_ in a 1:1 mixture of acetone and ethylene glycol solution. To identify the composition and resolution of the Tb(acac)_3_ stripes formed on the polymer surface, a UV laser (355 nm) and CCD connected to LCM was used to carry out spectroscopy and imaging studies. As Tb(III) emission starts from 480 nm and extends up to 628 nm, the signal from 376 nm to 464 nm was used for the polymer imaging (Fig. 5h). The high intensity PL peak at 545 nm (^5^*D*_4_ →^7^*F*_5_) corresponding to Tb(III) was used to imaging Tb(III) stripes. The overlay of the polymer and Tb(III) images (Fig. 5e and 5f) reveals the formation of cyan emitting stripe pattern on the blue emitting polymer surface (Fig. 5g).

**Figure 6:**
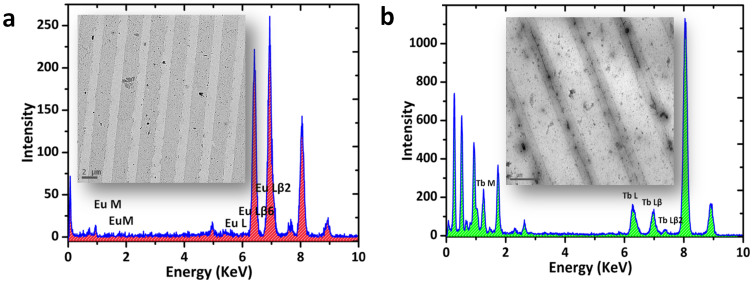
EDS data of the patterns formed on polymer film exhibiting a) Eu and b) Tb peaks, respectively. The inset show their corresponding TEM images of Eu(tta)_3_ and Tb(acac)_3_patterns formed from a polymer film coated on carbon coated TEM grids.

To investigate the stripes formed on polymer surface by using TEM, a carbon coated grid (mesh size : 200) was spin coated with the polymer followed by CP with Eu(tta)_3_ and Tb(acac)_3_ inks, individually. Bright-field TEM investigations of the Eu(tta)_3_ and Tb(acac)_3_ coordinated μ-CP patterns clearly displayed, formation of a series of 1D stripes corresponding to the metal complexed polymer (dark gray areas) and pure polymer (light gray areas) (insets of Fig. 6a and b, respectively). Additionally, the energy dispersive X-ray spectroscopic analyses (EDS) clearly demonstrated the presence of Eu and Tb complexes in the dark patterns displaying characteristic peaks (Fig. 6a and b, respectively).

After successful formation of metal coordination driven stripes on 2D polymer surface, we thought to extend the CP method to build wood-pile like stacked (yet very thin) 3D cross-stripes comprising Eu(tta)_3_ and Tb(acac)_3_ complexed polymer regions displaying multiple color pixel groups. It was also anticipated that this 3D patterning may generate square shaped white color emissive areas within each pixel group, due to overlay of three primary colors, blue from polymer, red and green from Eu(III) and Tb(III) ions, respectively. For this, a sample containing dried stripes of Tb(acac)_3_ coordinated onto a polymer a film was used as a substrate.

**Figure 7:**
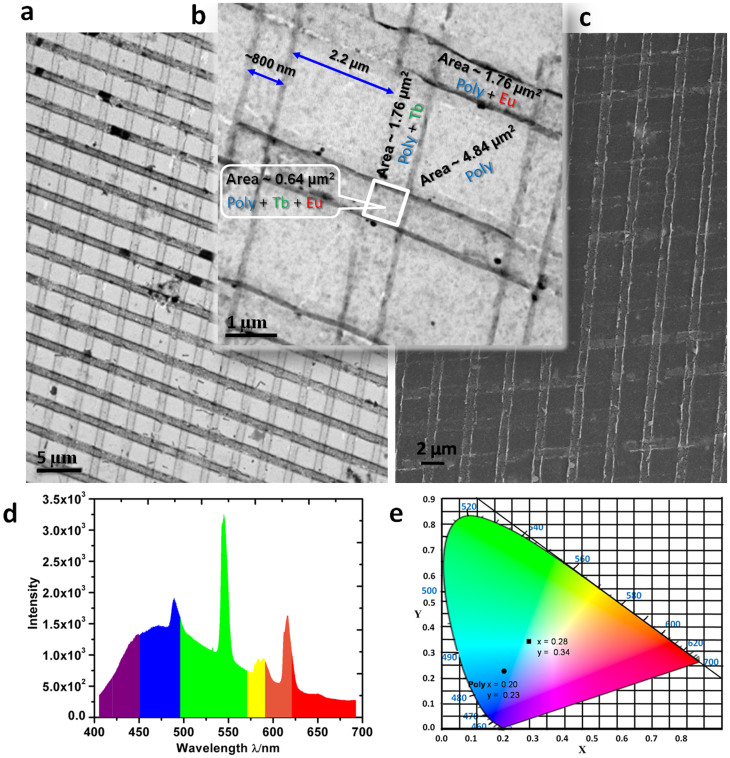
Hierarchical multicolor matrix pixels. (a) and (b), TEM image of Eu(tta)_3_ and Tb(acac)_3_ cross stripes formed on polymer (**Poly**) surface, in low and high resolutions, respectively. (c), FESEM image of the cross stripes. (d), Average emission spectrum of cross stripes obtained from an area of 13 × 3 *μ*m^2^ using a 355 nm UV laser scanning. (e), The corresponding CIE diagram is shown (black square) together with polymer (black circle).

To create cross-patterns, at first the direction of the Tb(III) stripes was determined by using a confocal optical microscope. Subsequently, over the Tb(III) patterned polymer film, another stamp inked with Eu(tta)_3_ was printed at an angle of ~90°. TEM and FESEM examinations of the pattern formed on a carbon coated TEM grid clearly demonstrated the formation of cross stripes, comprising Eu(III) and Tb(III), patterned over a large surface area (Fig. 7a and c, respectively). Figure 7b shows a close up view of the multicolor emitting cross stripes. The approximate pixel areas of the white square (Tb + Eu + Poly), pink rectangle (Tb + Poly) or purple rectangle (Eu + Poly) and the polymer square are ca 0.64 μm^2^, 1.76 μm^2^, and 4.84 μm^2^, respectively (Fig. 7b). Selective high resolution energy dispersive spectroscopic (EDS) line mapping of the Eu(III) and Tb(III) stripes exhibited corresponding metal ion peaks in the respective spectra (Fig. S4 b and c). Further, the EDS mapping of the whole pink square area (Tb + Eu + Poly) indicated the presence of both Eu(III) and Tb(III) ions, confirming the overall printing resolution of the cross pattern (Fig. S4 d–f). To verify the emissive properties of Eu(III) and Tb(III) cross-patterns on the polymer film, a selected area of the film was scanned by using a 355 nm laser and the obtained spectrum showed emission from 400–700 nm (Fig. 7d). The corresponding CIE diagram has values of *x* = 0.28 and *y* = 0.34 which is very close to white light, the pure polymer displayed the values in the blue region, *x* = 0.20 and *y* = 0.23 (Fig. 7e).

## Conclusions

In this work we have introduced an unprecedented coordination chemistry approach to create useful multicolor matrix pixel group below ~3 μm resolution using the conventional contact printing technique. To implement our strategy, we have synthesized a novel homopolymer containing tridentate bispyrazolylpyridine repeating units. The polymer thin film spin coated on a clean glass slide acts as a surface for reactive contact printing of luminescent metal ions via coordination chemistry. We have been able to create high resolution pixels consist of four colors (primary colors and white) in the nano/micro space from the cross stripes. We foresee that this innovative fabrication methodology to create multi-colour light emissive layers might be of interest for applications such as LEDs^23^,^24^,^25^.

## Methods

^1^H and ^13^C NMR spectroscopic data were recorded on a Bruker DPX 400 spectrometer; chemical shifts (δ) are expressed in parts per million relative to solvent proton as internal standard (CDCl_3_-d_1_ = 7.26 ppm). LC mass spectrometry was performed on Shimadzu LCMS-2010A mass spectrometer. IR spectra were recorded on JASCO FT/IR-5300 or Nicolet 5700 FT-IR. Elemental analysis was recorded on a Thermo Finnigan Flash EA 1112 analyzer. Spin coating was done on a glass substrate using Laurell TECHNOLOGIES CORPORATION Model WS-400B-6NPP/LITE/8K Spin-coater. UV-Visible absorption and fluorescence spectra were recorded on a spectrophotometer. The solid and liquid state absorbance spectra were collected from a Shimadzu UV-3600 spectrometer and a spectrofluorimeter (Horiba, JobinYvon) respectively. For fluorescence quantum yield measurements, solution of ligands in DCM was matched optically at an absorbing wavelength and then the quantum yield of quinine-sulfate in 0.1 M H_2_SO_4_ (Φ_f_ = 0.57, at 22°C) was used as reference. The molecular weights were determined by PSS-Win GPC (PSS) (pump: alliance GPC 2000) GPC equipped with an RI detector using a PL gel MIXED-B column (particle size: 10 mm, dimensions: 0.8 × 30 cm) calibrated against polystyrene standards. For thin-layer chromatography (TLC), silica gel plates Merck 60 F254 were used and compounds were visualized by irradiation with UV light.

### Atomic Force Microscopy Studies

AFM studies were carried out on NT-MDT Model Solver Pro M microscope using a class 2R laser of 650 nm wavelength having maximum output of 1 mW. All calculations and image processing were carried out by using NOVA 1.0.26.1443 software provided by the manufacturer. The images were recorded in a semi-contact mode using a super sharp silicon cantilever (NSG 10_DLC) with a diamond like carbon tip (NT-MDT, Moscow). The dimension of the tip is as follows: cantilever length = 100 (±5) μm, cantilever width 35 (±5) μm, and cantilever thickness = 1.7–2.3 μm, resonance frequency = 190–325 kHz, force constant = 5.5–22.5 N/m, chip size = 3.6 × 1.6 × 0.4 mm, reflective side = Au, tip height = 10–20 μm, and DLC tip curvature radius = 1–3 nm.

### Confocal Raman micro spectroscopy Studies

Raman spectra of the samples were recorded on a Wi-Tec alpha 200 confocal Raman spectrometer equipped with a Peltier-cooled CCD detector. Using a 600 grooves/mm grating BLZ = 500 nm, the accumulation was 10 and integration time was typically 0.5000 s. A He-Ne laser operating at 633 nm was used as an excitation source for the Raman scattering. All measurements were performed in air.

### PL imaging of stripes

The imaging for the Eu(tta)_3_complexed polymer were carried out using a backscattering (reflection mode, B-LCOM) laser confocal optical microscope of the WiTec alpha 300 SNOM instrument. The Nd:YAG laser operating at 532 nm (maximum output power is 40 mW) was used for the excitation of the stripes (spot size ~680 nm). In the backscattering mode experiment, a 100 × objective lens was used for the local excitation and for the collection of Raman and PL photons from polymer and Eu(tta)_3_ complex respectively. The collected signal was sent to a CCD detector through a multimode optical fiber of diameter 100 μm (core). The spectrum was recorded using a 532 nm long pass edge filter (LPEF) and a 300 grooves/mm grating BLZ = 500 nm, with a 0.1 s integration time. All measurements were carried out under ambient conditions.

The imaging for the Tb(acac)_3_ complexed polymer was carried out using a transmission mode laser confocal optical microscope (T-LCOM) facility of the WiTec alpha 300 SNOM instrument. UV laser operating at 355 nm (maximum output power is 7 mW) was used for the local excitation of the stripes by upright microscope using a 40 × UV objective (NA:0.6). The collection of PL photons was carried out through bottom microscope (kept below the sample) by using a 60 × visible range objective (NA:0.8). The collection was done in such a way that the illumination source (40×) and the collection unit (60×) were kept at fixed confocal position and the sample was kept in between the objectives on a piezo-scan stage. The collected PL signal was sent to a CCD detector through a multimode optical fiber of diameter 100 *μ*m (core). The PL spectrum was recorded using a 300 grooves/mm grating BLZ = 500 nm, with a 0.31 s integration time. Scanning was done on a 30 × 30 μm^2^ area. Total 120 × 120 spectra were collected to form 120 lines and each line was composed of 120 points to make a virtual image. All measurements were carried out under ambient conditions. Imaging for the cross stripe was done for an area of 13 × 3 *μ*m^2^ using a 355 nm laser. The collected PL photons were used to form an average spectrum. Converting the spectrum to the CIE coordinates shows the X and Y values are 0.28 and 0.34 respectively which are very close to the white light (X = 0.33, Y = 0.33).

### Solid state UV-Vis absorption and fluorescent studies

Diffused reflectance spectra were recorded for **M**, and **D** and the reflectance was converted to absorbance by following a Kubelka–Monk function^20^. For the reflectance measurement the solid sample was mixed with BaSO_4_ and mounted on a sample chamber. For the fluorescent measurement very thin KBr films of the sample **M** and **D** were prepared and mounted diagonally in a cuvette. The polymer was readily processed into a homogeneous film of good optical quality by spin-coating from CHCl_3_ at 6000 rpm on clean quartz substrates. The polymer coated substrate was used for the direct absorbance and fluorescent measurement.

### Synthesis of dimer (D)

In a 100 mL flask containing DMSO (20 ml), 2-(4-iodo-1H-pyrazol-1-yl)-4-octyl-6-(1H-pyrazol-1-yl)pyridine (**1**) (2.2 g, 4.869 mmol), bis(pinacolato)diboron (1.24 g, 4.896 mmol), K_2_CO_3_ (2.030 g, 14.69 mmol), and Pd(PPh_3_)_4_ (0.282 g, 5 mol %) were taken. The solution was stirred at 80°C under N_2_ atmosphere until the starting material was disappeared as monitored by TLC. The catalyst was removed by filtration and the residue was washed with CHCl_3_. The filtrate was cooled to room temperature and then washed with deionised water (5 × 50 mL). The organic layer was separated and dried over Na_2_SO_4_, filtered, and was evaporated in vacuo. The obtained light yellow solid residue was washed with MeOH (10 ml) to remove colored impurities to get white colour NMR pure compound of **D**. Alternatively, purification of the yellow solid residue was also can be done by column chromatography (2:3 n-Hexane:DCM) on activated silica gel to get an analytically pure white color powder of **D**. (1.16 g, 74%yield).^1^H NMR (400 MHz, CDCl_3_, 25°C): δ8.62 (s, 2H), 8.55 (d, 2H, J = 2.4 Hz), 7.76 (d, 4H), 7.73 (s, 2H), 7.65 (s, 2H),6.5 (m, 2H), 2.76–2.72 (t, 4H, J = 8 Hz), 1.74–1.70 (t, 4H, J = 8 Hz), 1.37–1.27 (m, 22H), 0.89–0.86 (6H). ^13^C NMR (100 MHz, CDCl_3_, 25°C): δ158.5, 150.1,149.8,142.3, 140.3, 127.3, 123.3, 115.5, 109.6, 109.4, 107.9, 35.9, 31.8, 30.2, 29.4, 29.3, 29.2, 22.6, 14.1. FT-IR (KBr): 3128.8, 2958.9, 2926.0, 2849.3, 1742.5, 1715.1, 1621.9, 1567.1, 1528.8, 1452.1, 1391.8, 1260.3, 1189.0, 1046.6, 969.9, 915.1, 860.3, 789.0, 761.6, 646.6, 602.7 cm^−1^; UV/Vis (CHCl_3_): absorbance = λ_max_317 nm and emission λ_max_ = 344 nm; LCMS (m/z): calcd. for C_38_H_48_N_10_, 644.4063; found, 645.45; HRMS (m/z): [M + Na]^+^calcd. for C_38_H_48_N_10_, 644.4063; found 667.3974; analysis (calcd., found for C_38_H_48_N_10_) C (70.78, 70.61), H (7.50, 7.58), N (21.72, 21.62); CCDC No. 992513. M.p.: 141°C.

### Synthesis of polymer (Poly)

Bis(cyclooctadiene)nickel(0) (0.342 g, 2.4 equiv), cyclooctadiene (0.153 mL, *d* = 882 mg/mL, 2.4 equiv), and 2,2′-bipyridine (195.48 mg, 2.4 equiv) were dissolved in a solution of dry toluene (5 mL) and dry N,N-dimethylformamide (5 mL) in a Schlenk flask within a glove box. The mixture was heated at 60°C with stirring under argon for 20 min to generate the catalyst, and then a solution of the 2,6-bis(4-iodo-1H-pyrazol-1-yl)-4-octylpyridine **2** (300 mg, 0.521 mmol) in dry toluene (5 ml) was added. The reaction was heated at 80°C for 2 days. Then a toluene solution (4 ml) of 2-(4-iodo-1H-pyrazol-1-yl)-4-octyl-6-(1H-pyrazol-1-yl)pyridine (70.29 mg, 0.156 mmol) was added and the reaction mixture was heated at 80°C for an additional 12 h. The crude reaction mixture was then poured into a mixed solution (100 mL) of methanol and concentrated hydrochloric acid (1:1) and stirred for 12 h. The precipitated white solid was filtered and dissolved in THF (10 ml) and added drop wise to methanol (200 ml). The resulting solid was filtered off and subjected Soxhlet extraction for 2 d in acetone (130 ml) followed by methanol (volume 130 ml). The insoluble solid residue was redissolved in THF (10 ml) and precipitated from methanol (180 ml). The precipitate was washed with methanol (10 ml) and air dried to get polymer in 126 mg yield.^1^H NMR (500 MHz, CDCl_3_): δ 9.16 (s), 8.65–8.85 (end group), 7.89 (s), 7.6–7.8 (end group), 7.46 (s), 6.5–6.6 (m, end group), δ 2.83–2.80 (m), δ 1.77–1.30 (m), 0.91 (m); FTIR (KBr disc): 3408, 3107, 2959, 2926, 2860, 1616, 1573, 1529, 1447, 1392, 1184, 1041, 975, 921, 860, 789, 652, 603 cm^−1^; UV/Vis (CHCl_3_): absorbance = λ_max_ 316 nm and emission λ_max_ = 366 nm; GPC (THF): *M*_n_ = 7.39 kDa (number average), *M*_w_ = 14.27 kDa (weight average) and PDI = 1.93 (poly dispersity index).

## Author Contributions

The manuscript was written through contributions of all authors. All authors have given approval to the final version of the manuscript. R.C. conceived the research idea. S.B. performed synthesis and patterning work under the supervision of R.C., M.B. and K.M. S.B. and A.M.M. performed the imaging experiments under the supervision of R.C. and involved in the data analysis and discussions. S.B., K.M. and R.C. wrote the manuscript.

## Supplementary Material

Supplementary Information Supplementary Information

## Figures and Tables

**Figure 1:**
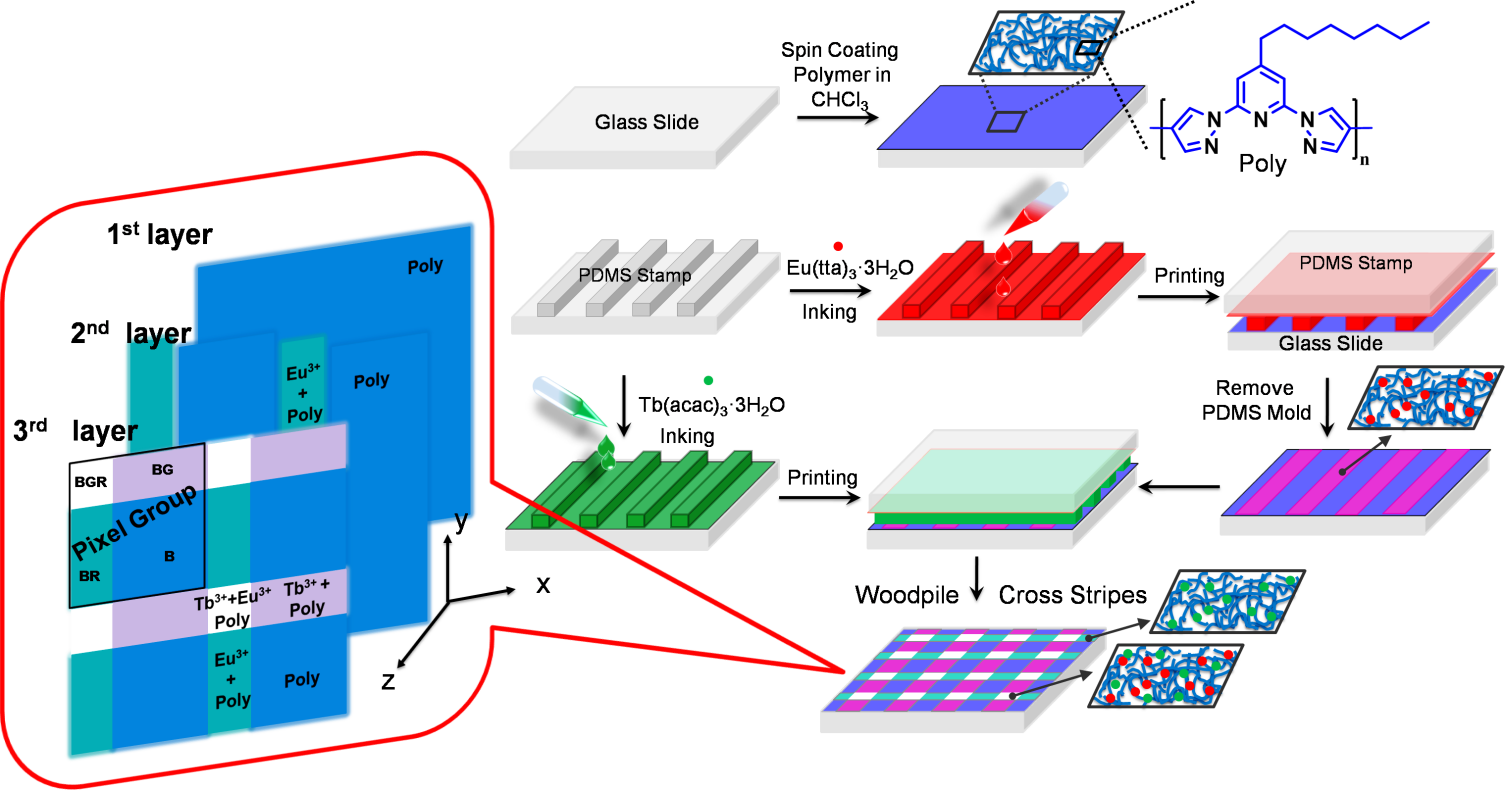
Fabrication Strategy. Graphical representation of a hierarchical reactive contact printing process leading to multicolor matrix pixels.
