# Supplementary material for: Hierarchical multicolor nano-pixel matrices formed by coordinating luminescent metal ions to a conjugated poly(4′-octyl-2′,6′-bispyrazoyl pyridine) film via contact printing
Source: Sci Rep. 2015 Feb 12;5:8406. doi: 10.1038/srep08406 (PMC4325338; doi:10.1038/srep08406)

Supplementary Information

Hierarchical multicolor nano-pixel matrices formed by coordinating luminescent metal ions to a conjugated poly(4’-octyl-2’,6’-bispyrazoyl pyridine) film via contact printing

Supratim Basak^1^, Md Ahamad Mohiddon^1^, Martin Baumgarten^2^, Klaus Müllen^2^,& Rajadurai Chandrasekar^1^*

^1^Functional Molecular Nano-/Micro-Solids Laboratory, School of Chemistry, University of Hyderabad, Prof. C. R. Rao Road, Hyderabad – 500046, India, ^2^Max Planck Institute for Polymer Research, Ackermannweg 10, D-55128, Mainz, Germany

Corresponding author: chandrasekar100@yahoo.com (or) rcsc@uohyd.ernet.in

**GENERAL EXPERIMENTAL METHODS:**

**Materials:** Citrazinicacid, (COCl)_2_, I_2_, trifloro acetic acid, tetramethylammonium chloride, [Pd(PPh_3_)_4_] and deuterated solvent CDCl_3_-d1was obtained from Aldrich. K_2_CO_3_, LiOH, Cu(I)I, PPh_3_ were purchased from Avra Synthesis, Hyderabad, India. THF, Triethylamine, benzene, dichloromethane, hexane, pet-ether, CHCl_3_ and methanol solvents were obtained from Finar Chemicals Limited, Ahmedabad, India. All solvent were used after distillation. Methanol, HCl and NaN_3_ obtained from Merck. MgSO_4_ and Na_2_S_2_O_3_ were purchased from SRL chemicals. Pvt. Limited, Hyderabad. For UV-Vis and Fluorescence measurements spectroscopic grade solvents were used.

| **SOLUTION (CHCl_3_)** | | | | | **THIN FILM** | | | |
| --- | --- | --- | --- | --- | --- | --- | --- | --- |
| Compound | Absorption λ_max_ (nm) | Excitation λ_ex_ (nm) | Emission λmax (nm*)* | *Φ_f_^a^* | Absorption λ_max_ (nm) | Emission λ_max_ (nm) | Excitation λ_ex_ (nm) | Optical band Gap /eV |
| **M** | 268, 301 | 301 | 330 | 37% | 244, 268, 301 | 351 | 301 | 4.1 |
| **D** | 253, 317 | 317 | 344 | 24% | 253, 320 | 361 | 320 | 3.87 |
| **Poly** | 258, 316 | 316 | 366 | 7 % | 227, 323 | 353, 366 | 323 | 3.83 |

Table S1: Spectroscopic data of the solution/solid state absorption and emission spectra of monomer, dimer, and polymer. The band gaps were calculated from the corresponding absorption edges.


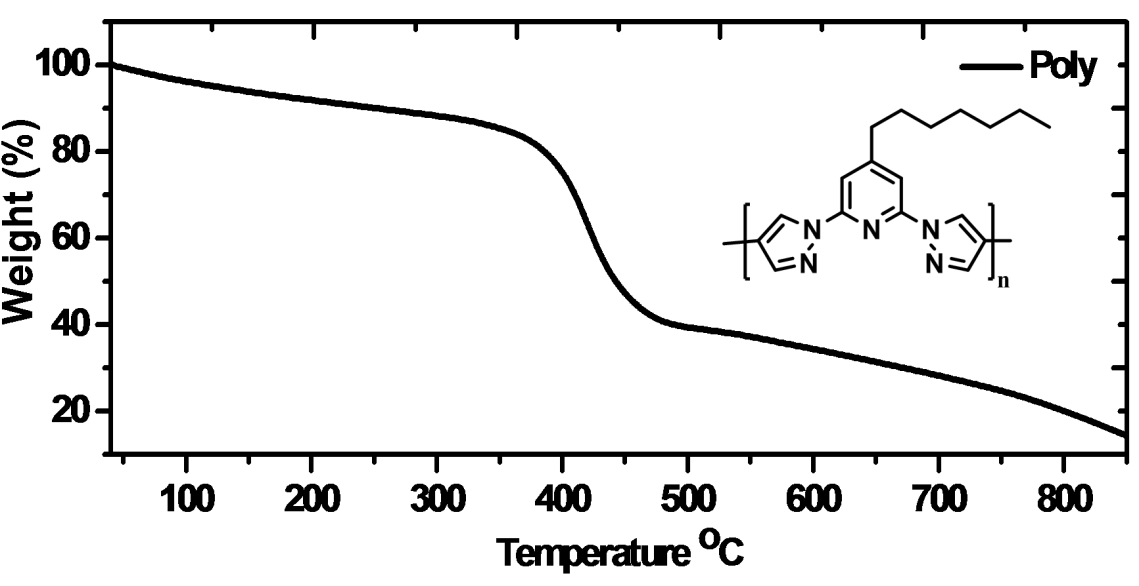


Figure S1.TGA of the Polymer (Poly)


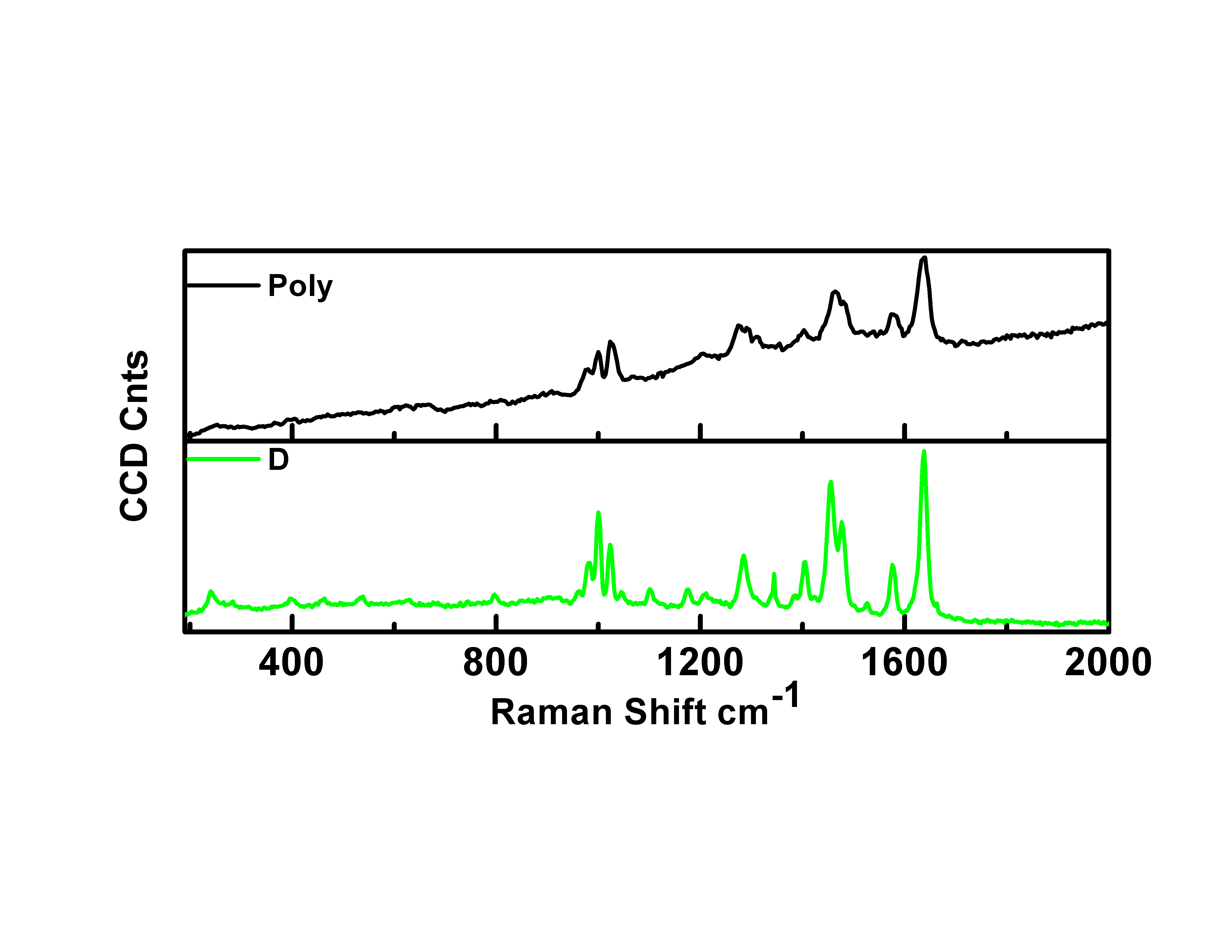


Figure S2. Comparison of the Raman spectra for bulk polymer (Poly) and dimer (D).


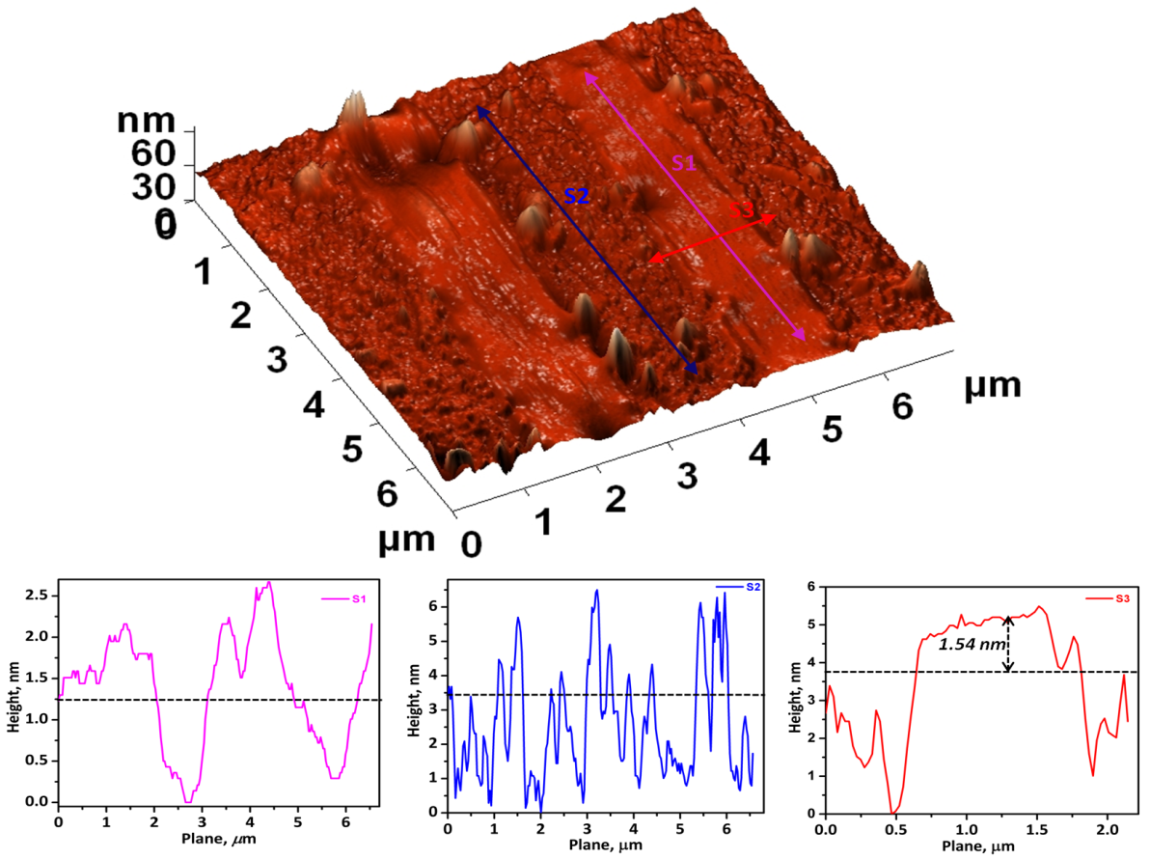


Figure S3. Tapping mode AFM image displaying the roughness profiles: along Eu(III) printed area ~ 1.25 nm (S1), and pristine polymer film ~ 3.5 nm (S2). The printed area is ~ 1.5 nm thick compared to the polymer film (S3).


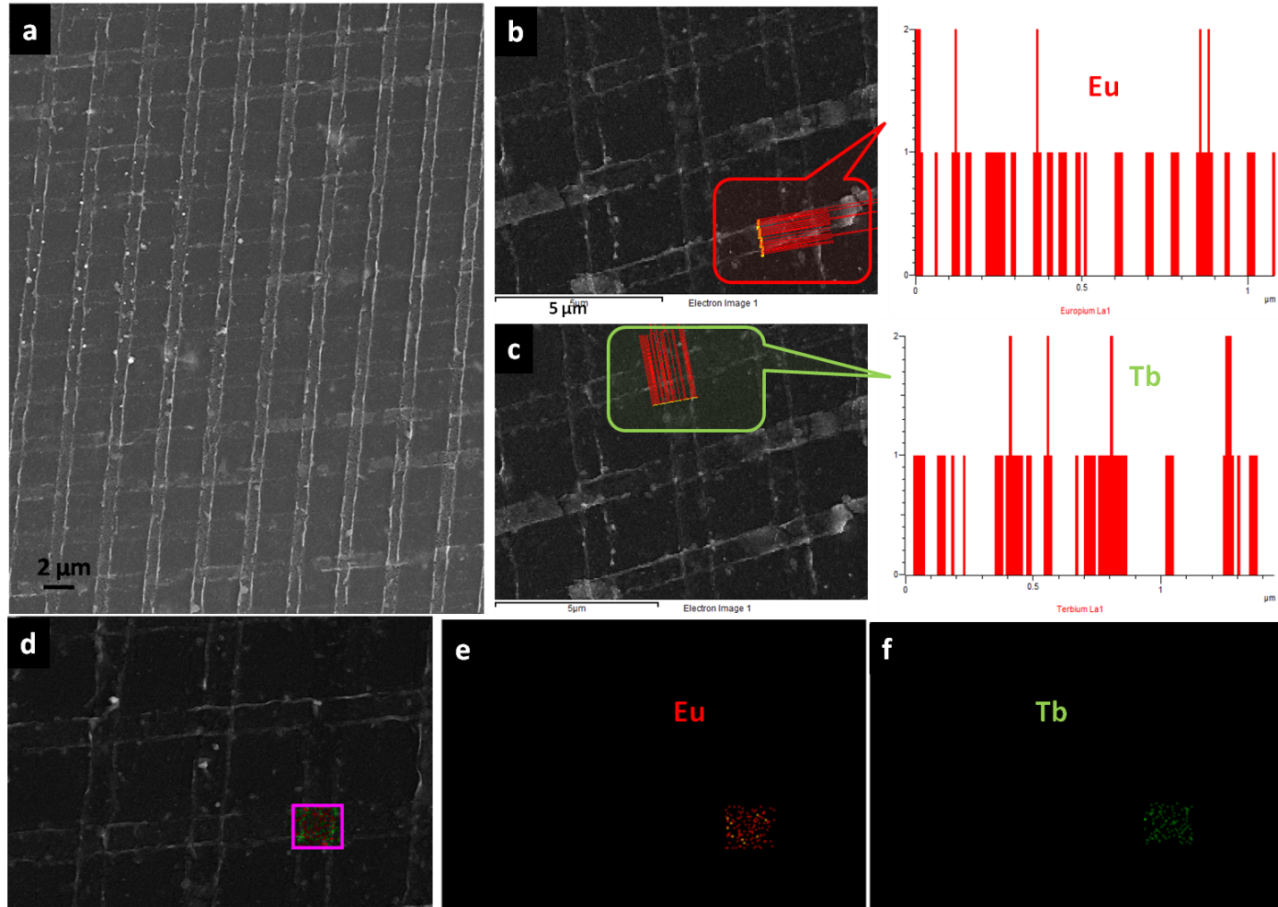


Figure S4. a) FESEM image of Eu(tta)_3_ and Tb(acac)_3_ cross stripes patterned reactively on a polymer (Poly) surface (scale bar is 2 μm). b and c) EDS elemental mapping of the Eu(tta)_3_ and Tb(acac)_3_ stripes indicating the presence of the corresponding ions. d) EDS mapping of a square area containing both Eu(tta)_3_ and Tb(acac)_3_, their corresponding elemental mapping is shown in Fig e and f, respectively.

CHARACTERIZATION DATA:


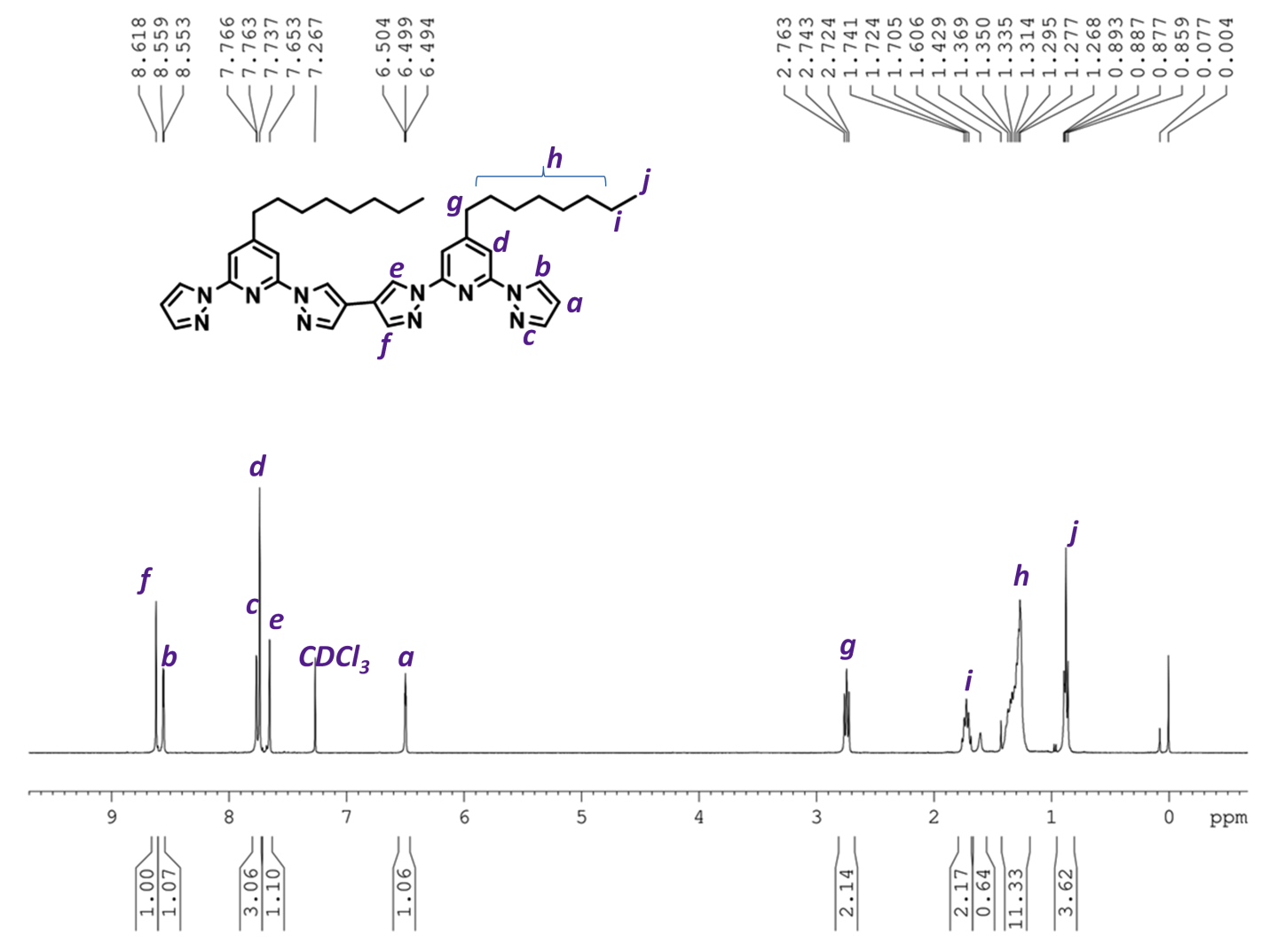


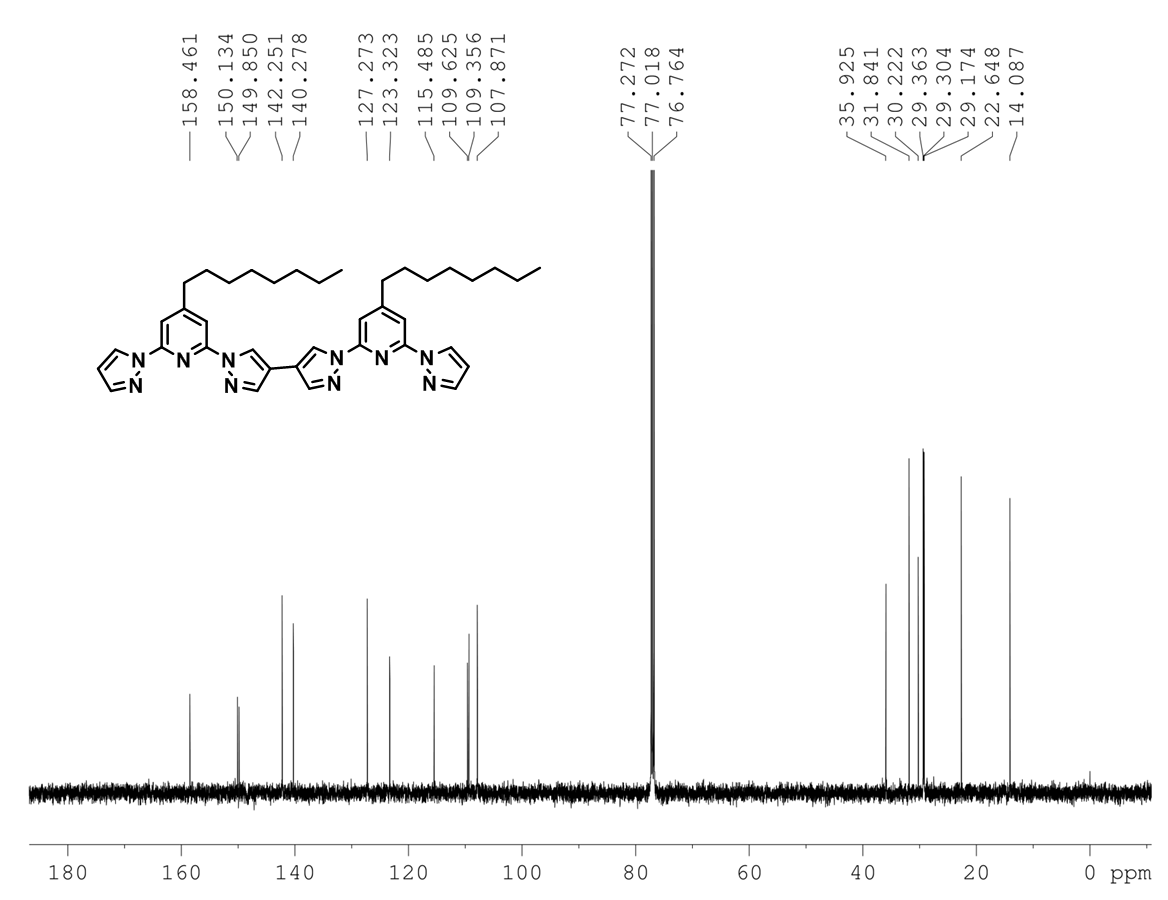


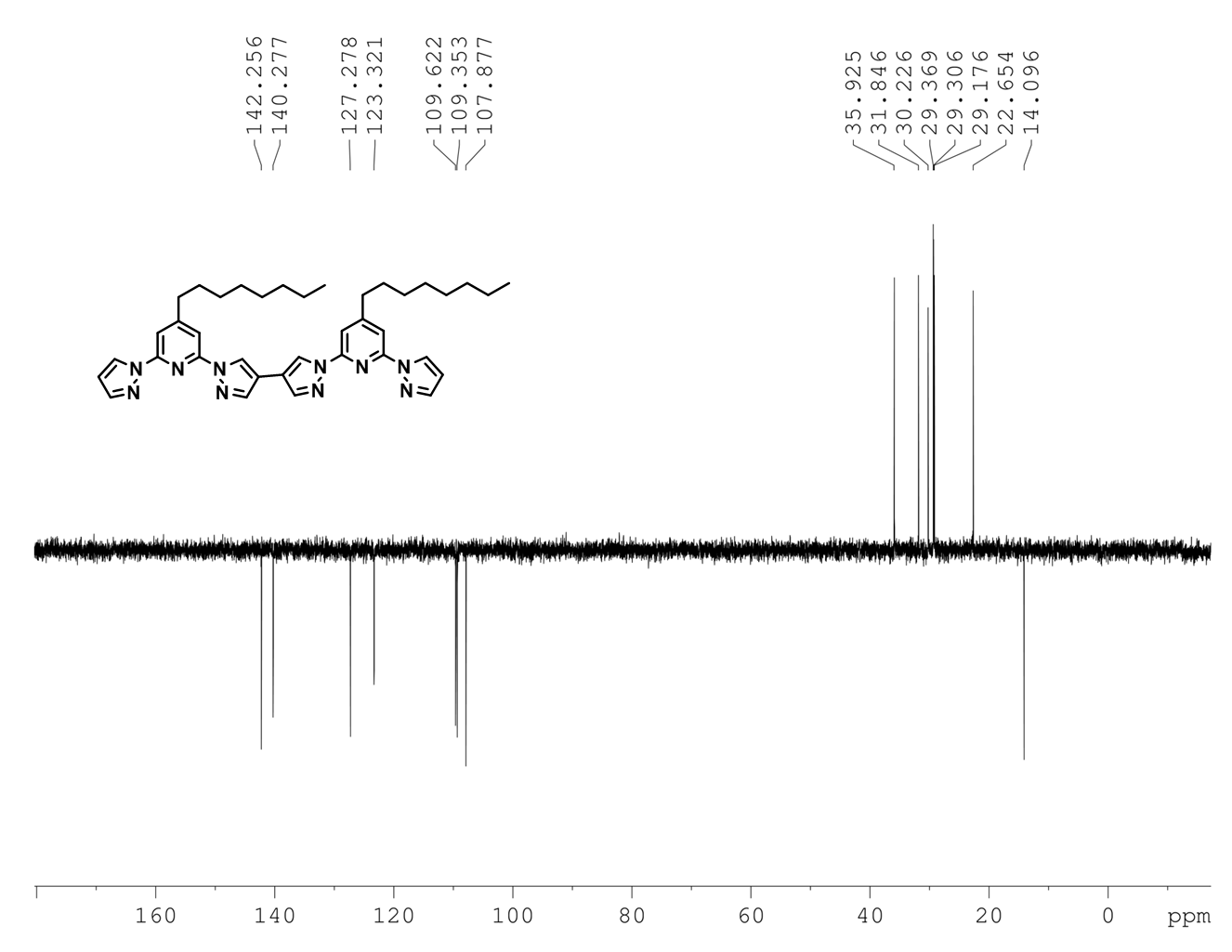


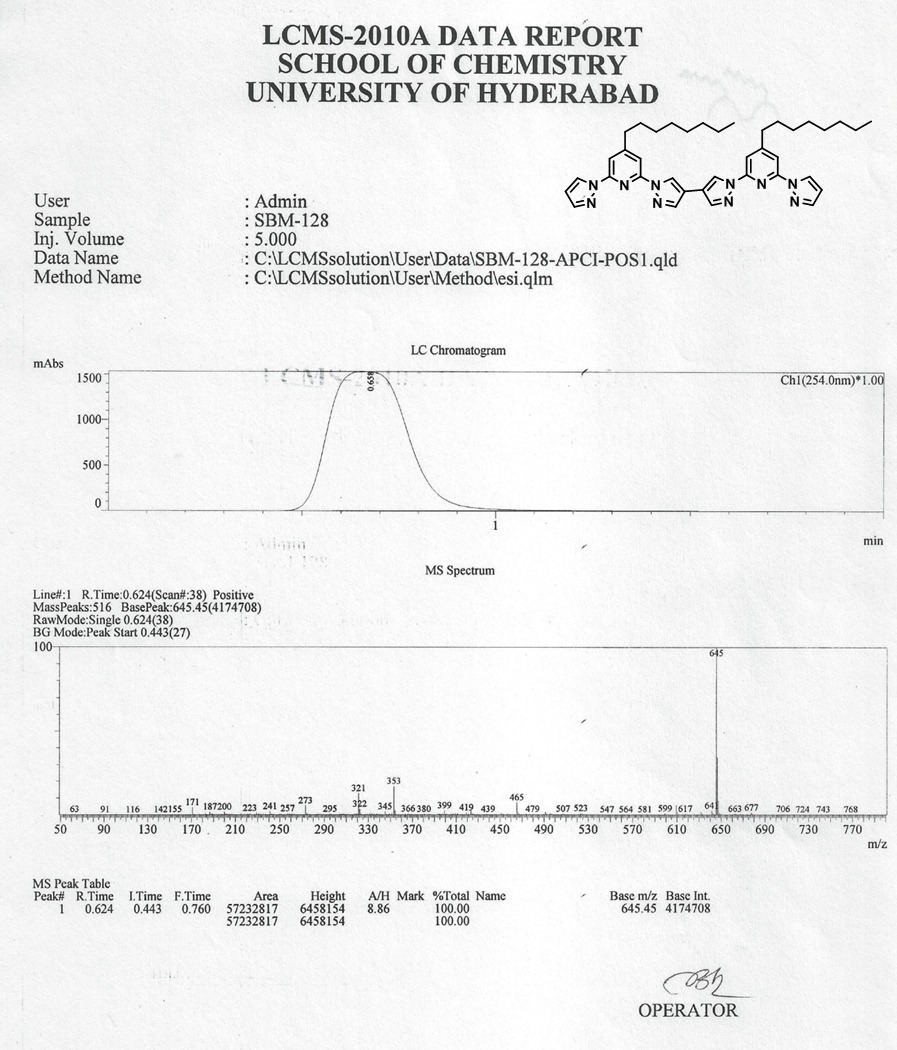


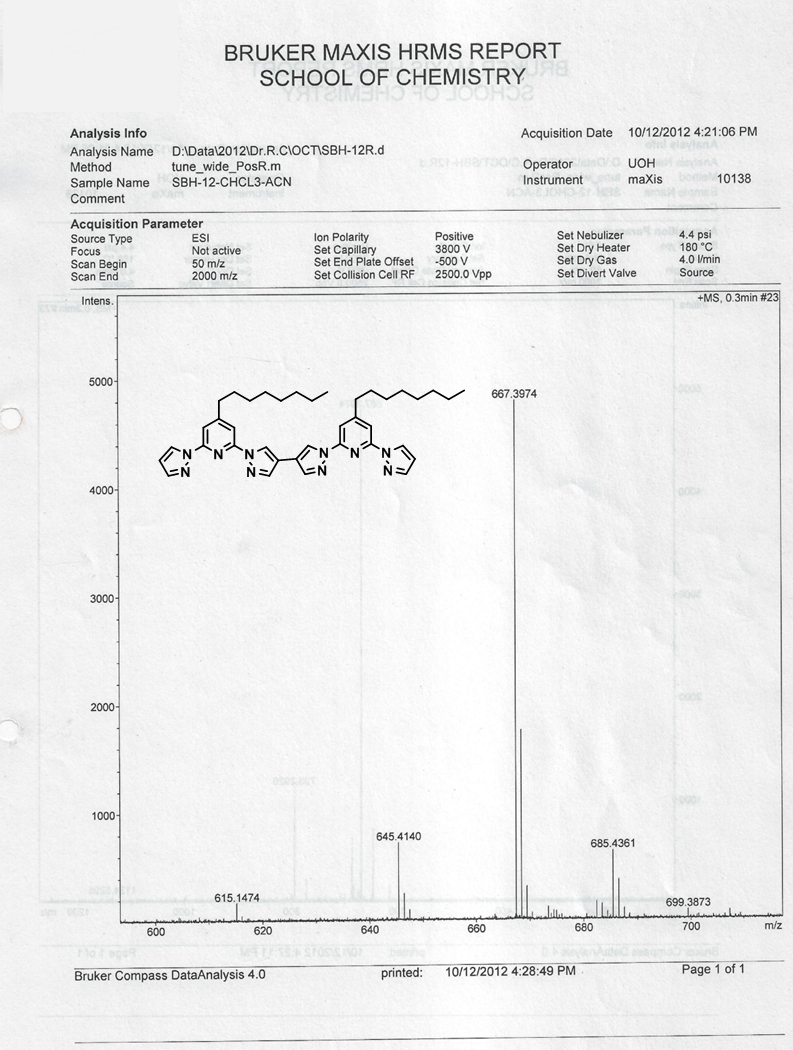


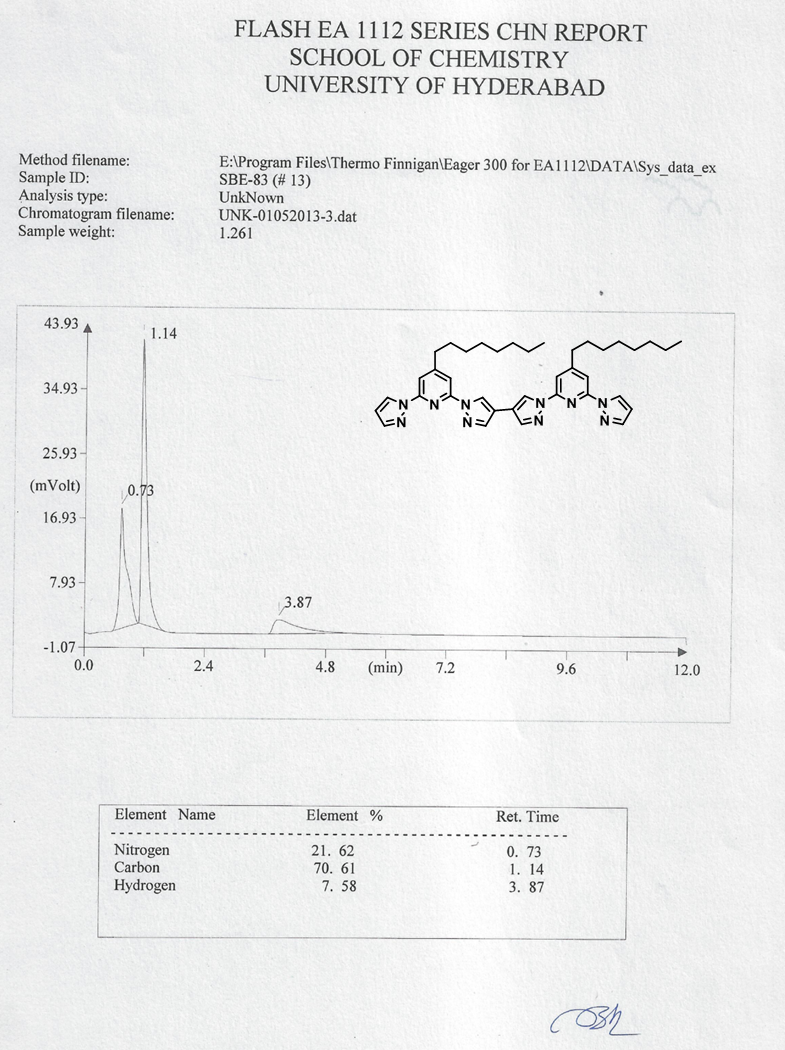


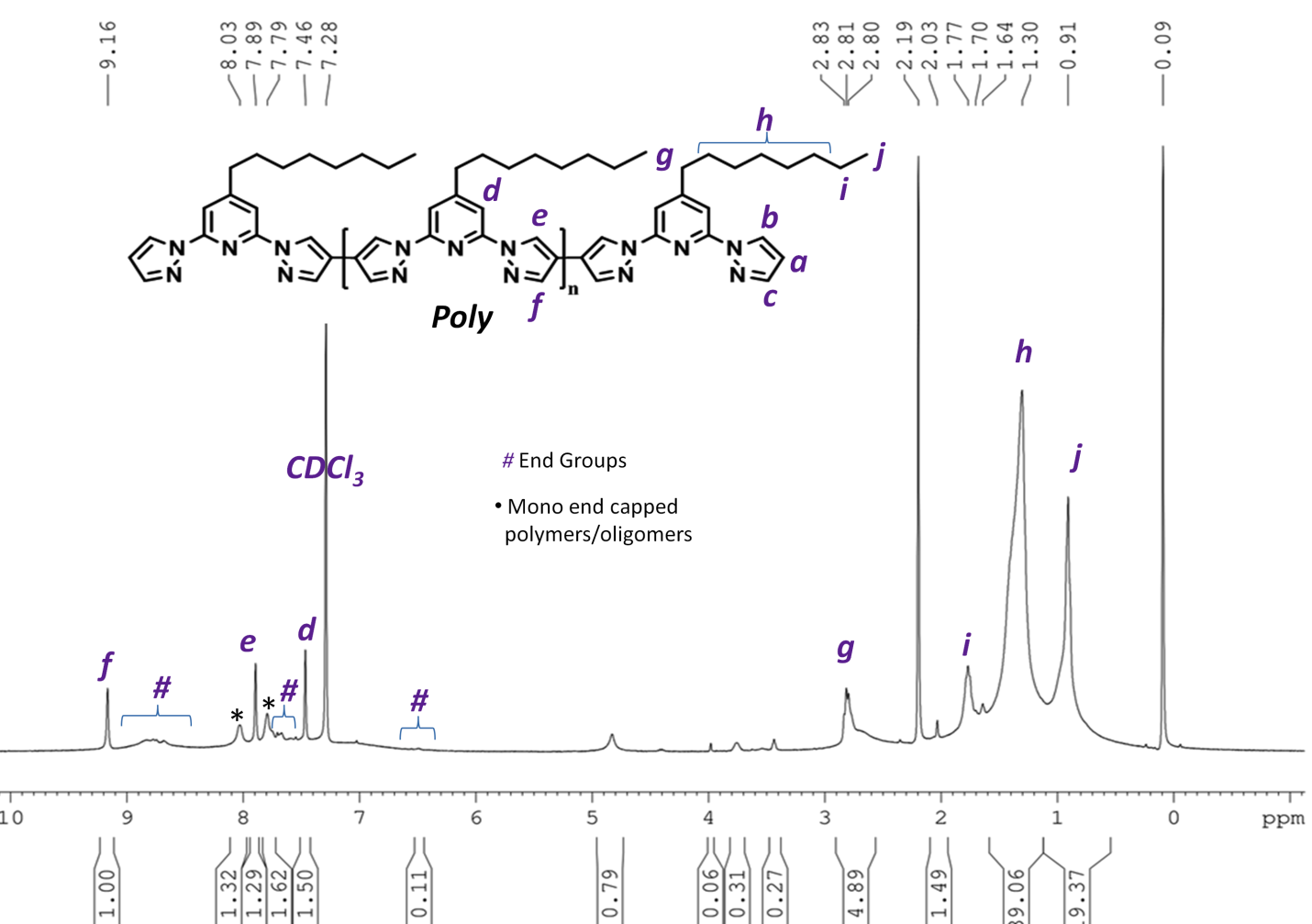

Supplement: Supplementary Information [file srep08406-s1.docx]
